# Barriers and facilitators of micronutrient supplementation among non-pregnant women of reproductive age in Johannesburg, South Africa

**DOI:** 10.1371/journal.pgph.0001310

**Published:** 2022-11-30

**Authors:** Takana M. Silubonde, Catherine E. Draper, Jeannine Baumgartner, Lisa J. Ware, Cornelius M. Smuts, Shane A. Norris

**Affiliations:** 1 Centre of Excellence for Nutrition, Faculty of Health Sciences, North-West University, Potchefstroom, South Africa; 2 SAMRC/Wits Developmental Pathways for Health Research Unit, Faculty of Health Sciences, University of the Witwatersrand, Johannesburg, South Africa; 3 Department of Nutritional Sciences, King’s College London, London, United Kingdom; 4 School of Health and Human Development, University of Southampton, Southampton, United Kingdom; Dow University of Health Sciences, PAKISTAN

## Abstract

The prevalence of anaemia among South African women of reproductive age (WRA) remains high at 39%. Multiple micronutrient supplementation (MMS) may be an effective strategy in the prevention and management of anaemia. Our aim was to understand facilitators and barriers to preconception MMS adherence and to explore perceptions and beliefs of MMS in the prevention and treatment of anaemia among non-pregnant WRA. This qualitative study was embedded in a preconception MMS intervention trial of WRA and was conducted in two phases. Phase one assessed the barriers and facilitators of MMS adherence. Individual interviews were conducted with the community health workers (n = 7) administering MMS, and with non-pregnant WRA (n = 25) participating in the trial. Phase two included four focus groups with participating WRA (n = 26), which further explored participants’ perceptions and beliefs of MMS provision and adherence, and strategies to improve adherence. The reported facilitators to supplementation were family support, interaction with the community health workers, easy access to MMS, and experienced benefits of MMS. Barriers to preconception supplementation included the lack of family support, the link of supplements to antenatal care, and the perceived lack of benefits of MMS. Participants reported negative associations of supplements with medication, individual and societal stigma around medication and challenges around the supplementation schedule. For successful preconception MMS interventions, young women, their families, and communities need to be convinced of the value of supplementation. Public health interventions utilising preconception supplementation will require specialised training for health care providers, targeted counselling materials and community household support

## Introduction

It is estimated that globally 33% of women of reproductive age (WRA) are anaemic [1]. A recent systematic review in South Africa reported that anaemia among WRA remains a moderate-severe public health concern with reported prevalence rates between 22% and 44% [2]. Though the causes of anaemia are multifactorial, it is estimated that 50% of anaemia cases are as a result of iron deficiency [3]. Data in the same study population, in which the current study is being conducted, reported that the prevalence of anaemia, iron deficiency and iron deficiency anaemia among (18–25-year-old) WRA was 39%, 38% and 22%, respectively [4]. As such, women are likely to enter pregnancy with less than optimum iron stores, which may have detrimental effects on them and their offspring including increased risk of miscarriages, stillbirths, prematurity and low birth weight, as well as impaired physical and neurological development of the offspring [5–7]. Reduction of anaemia is a key target of the World Health Assembly Global Nutrition Targets for 2025 and the Sustainable Development Goal (2030) [8], but South Africa has made no progress towards achieving this [2].

In most settings, the most cost-effective strategy to prevent and reduce anaemia includes food fortification, dietary modification, and oral iron supplementation. As recommended by the World Health Organisation [8], the South African Government has policies in place to ensure that women receive iron supplements as part of the standard antenatal care, to alleviate and prevent anaemia in pregnancy [9]. However, in spite of this, anaemia prevalence in pregnancy remains high. Hoque et al. reported that 52% of pregnant women were anaemic [10]. It is also well documented that the majority of pregnant women (54%) only get access to antenatal care in their second trimester [11, 12]. Hence, this intervention misses critical developmental processes occurring early in pregnancy. To ensure women enter pregnancy with sufficient iron stores a shift to preconception iron supplementation may be of benefit [13].

Recently, we showed the presence of inflammation and vitamin A deficiency were significant risk factors of anaemia in WRA [14]. Furthermore, Symington *et al*. [10], reported increased anaemia prevalence during pregnancy despite routine iron supplementation. Thus, intervening with iron alone may not prevent or treat anaemia, and administering high doses of iron to iron replete individuals may even be associated with adverse birth outcomes [10]. The use of multiple micronutrient supplements (MMS) with a lower dose of iron and other micronutrients has been recommended as a potentially more efficacious intervention [15].

The overarching aim of our study was to provide micronutrient supplements to women in the preconception phase, to ensure that they may enter pregnancy with optimum iron stores. However, a preliminary investigation done by the research team revealed that women were unfamiliar with taking micronutrient supplements outside of pregnancy. Women also reported difficulty in taking the supplement, as they were not used to taking ‘medication’ regularly. It has been documented that negatively viewing supplements as medicine can be a barrier to supplementation [16]. Additionally, having to consume ‘pills’ for extended periods can negatively affect adherence [16]. However, data on challenges of adherence to nutrient supplementation research remains sparse. Therefore, to enhance the effectiveness of our intervention, we firstly aimed to understand facilitators and barriers to preconception supplementation adherence, and secondly, to identify strategies for improving supplementation adherence for anaemia treatment and prevention.

## Methods

The study was conducted in two phases and was embedded in a preconception health trial with WRA, which included administration of an MMS. Phase one consisted of individual interviews with both participants and community health workers (CHW) referred to as ‘Health Helper’ who administered the MMS as part of the trial. This phase was used to gain an understanding of the barriers and facilitators of MMS supplementation adherence. In phase two, focus groups were conducted with different trial participants to further explore their perceptions and beliefs of MMS provision and adherence.

### Study setting and participant recruitment

The study was conducted in Soweto, a historically disadvantaged urban area spanning two hundred km of Johannesburg. Soweto is characterised by rapid economic development, urbanisation, social mobility, and persistent inequality. Despite a rising middle class, Soweto still has high rates of unemployment, poverty and food insecurity [17]. Recruitment criteria for the ongoing trial are women aged 18–28 years, and residence in their home in Soweto for at least 3 months. Exclusion criteria are diagnosis of type 1 diabetes, cancer, or epilepsy; or not able or willing to provide written informed consent. Due to South Africa’s high prevalence of human immunodeficiency virus (HIV) infection (23% of women aged 15–49 years) [18], women who were HIV positive were included in the study for the sample to be a better representation of the general population.

### Study design

The trial is being conducted at the South African Medical Research Medical Council (SAMRC) Developmental Pathways to Health Research Unit (DPHRU), located within the Chris Hani Baragwanath Academic Hospital. Briefly this is a randomised control trial based on the baseline assessment of haemoglobin status measured in capillary blood using a calibrated Hb 201+ HemoCue system (HemoCue Johannesburg, South Africa). Anaemic women in the intervention arm received a daily MMS containing 27 mg iron and monthly contact by CHW to promote physical and mental health. Non-anaemic women in the intervention arm received MMS containing 27 mg iron twice a week and monthly contact. All intervention participants received a calendar to mark each time they took supplements. Women in the control group received monthly telephone calls to support life skills development (21). However, if their Hb at enrolment was below 12 g/dL, they were referred to the clinic for treatment based on current standard of care.

For phase one, twenty-five intervention participants were purposively selected from the database of women in the existing trial and contacted telephonically to request their participation in an individual interview. All CHW (n = 6) and the driver who delivered supplements were included in the sample and were invited to participate in an interview. For phase two, 26 intervention participants were purposively selected and invited to participate in focus groups.

### Ethical considerations

All participants gave written informed consent before taking part in the study, and ethical approval for the qualitative study and trial was granted by the Human Research Ethics Committee (Medical) at the University of the Witwatersrand (M190449, M1811111).

## Data collection

### Phase 1: Individual interviews

Interview guide questions were developed in English for the participants and their CHWs by three researchers (CD, LW, TS). The major domains of the interviews centred around barriers and facilitating factors for MMS adherence. A trained female research assistant, fluent in participants’ languages, conducted the participant interviews in English, with the flexibility for participants to use vernacular languages. Due to COVID-19 restrictions at the time, these interviews were conducted telephonically, and audio recorded; they ranged in length between 10 to 20 minutes. The CHW and driver interviews were conducted in person by TS (female PhD student who has a working relationship with the trial team) in English.

### Phase 2: Focus groups

**Fig 1:**
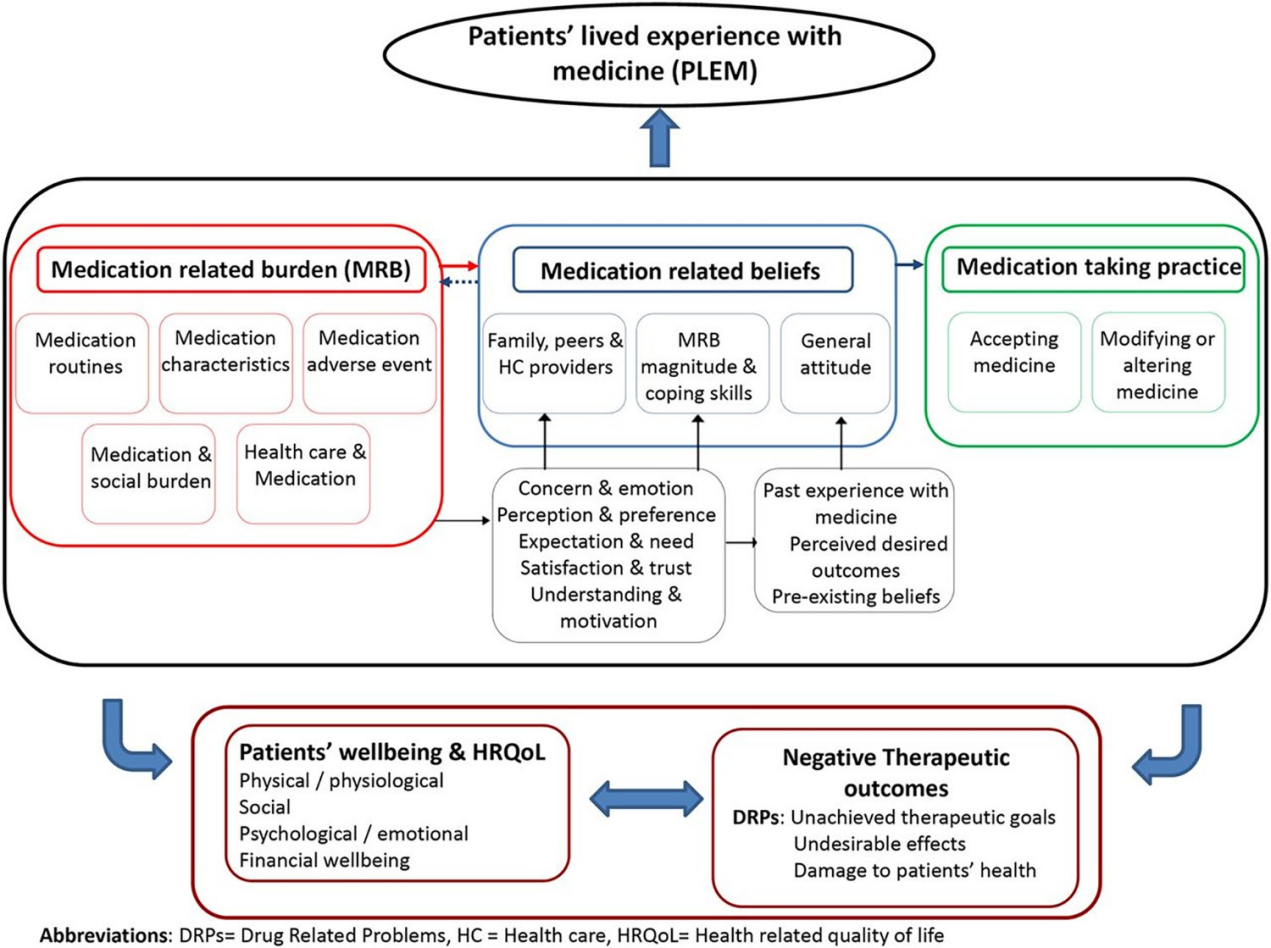
Conceptual framework on patients lived experience with medication [17]. Abbreviations: DRPs = Drug Related Problems, HC = Health care, HRQoL = Health related quality of life.

The individual interviews were analysed, and learning from the individual interviews assisted the formulation of focus group questions that were also informed by the conceptual model of patients’ lived experience with medicines [16] (PLEM, Fig 1). The main domains of the focus groups were (i) Medication related burden (which covered issues concerning medication taking routines, medication characteristics, medication adverse events and the healthcare system and medication), and (ii) Medication related beliefs (which discussed participant, family and peers’ perceptions, emotions, motivation and beliefs around medication and anaemia). Four focus group discussions (FGDs) were conducted between October and November 2021. Each FGD was composed of 6–8 participants, and two multilingual research assistants facilitated the FGD discussions using a semi-structured discussion guide. The FGDs were audio recorded, one research assistant conducted observations and took intensive notes as a backup for the audio files. The discussions, which took 60–80 minutes, were conducted in English with the flexibility of the participants to use vernacular languages.

## Data analysis

Interview and FGD recordings were transcribed verbatim, translated into English when necessary, and checked for accuracy. Data were thematically analysed manually by TS using a largely deductive approach [19]. For the phase one interviews, the interview guide formed the basis of an initial framework, and this was further developed to encompass the following themes: participant barriers and facilitators of supplementation adherence; CHW barriers and facilitators of supplementation adherence. For the FGDs in phase 2, drawing on the framework and conceptual model mentioned above, the FGD guide questions formed the basis of the framework, that also considered the phase one interview findings. This framework included the following themes: individual level beliefs and perceptions of anaemia and medication, societal norms and practices and medication related beliefs, health care provision, access, supply, and circumstances of daily life. After the stage of familiarisation with the data, codes were developed based on these themes. The next step involved searching for themes in the transcripts, and continuously reviewing and refining themes. Once coded, sections of text were summarised for each theme and illustrative quotes extracted.

## Results

### Phase 1: Interviews

#### Participant facilitating factors for supplementation adherence

Five subthemes were identified when participants discussed facilitating factors supplements adherence: counselling of CHW; benefits of supplements; family support; knowledge of anaemia; and access to supplements.

It is clear from the data (illustrative quotes presented in Table 1) that prior to the intervention, most young women had little to no knowledge on the need for women to take MMS during the preconception phase. Counselling at the onset of supplementation on the benefits of supplementation by the CHW was crucial to women agreeing to take the MMS. After counselling, women reported that they understood the purpose of supplementation, were willing to participate in the intervention, and trusted that the MMS would benefit them.

**Table 1:** Participant quotes regarding barriers and facilitators to supplementation adherence.

Barriers	Quotes
**Knowledge of anaemia**	*‘I thought I’d suffer complications related to those supplements; I just didn’t trust them’*
	*‘Uh I don’t have any bad experiences about pills*, *but there are…the iron supplements I’ve never*, *ever taken them…*. *In my entire life*, *so it was my first experience*.*’*
**Lack of experienced benefits**	*I can’t tell*, *I am not sure*. *Because it does not have any effects*, *because as I said I did not know that I have iron deficiency*, *so I don’t know whether it has been cured or not*.*’*
**Experienced side effects**	*‘For me I stopped taking them because when I was taking them*, *I started getting these huge pimples and my face changed […] I stopped taking them immediately*. *It was bad*, *so my face was bad for three months*, *you know it was so sad because my skin is usually so clear*. *[…] Now I don’t want to drink any medication for my skin because I feel like*, *if I take another supplement what if it does worse than the one*, *I took now*.*’*
**Family support**	*‘My granny would ask if I trust the people that gave me this supplement and then she didn’t trust them’*
**Facilitators**	
**Knowledge of anaemia**	*Well*, *she told me those supplements were to boost my iron*, *not that I have iron deficiency*, *but to boost my iron’*
**Perceived benefits**	*I have been having a lot of energy*, *ever since I started the supplements everything is okay now*, *I no longer sleep during the day*, *I don’t find myself always being tired*, *ja*.*’*
**Family support**	*‘‘…my mom is close by…*. *She’s the one who’ll remind me that I need to take the pills; I asked her to remind me if I forget*.*’*
**Counselling from CHW**	*‘Like I didn’t know what my iron was like and then she explained to me that it was like this and if I take supplements my iron would be alright*. *They are trying to not get you sick*.*’*
**Access to MMS**	*Oh yes*, *definitely I do appreciate that if I don’t have transport money to come to Bara to fetch the supplements*, *then what*? *So*, *I really appreciate them delivering at my doorstep*.*’*

Participants reported that experiencing benefits motivated them to continue with the MMS. Perceived benefits reported included: glowing skin, weight gain, feeling ‘good,’ a cessation of headaches, and having more energy. Most participants reported that after they explained the benefits of the MMS to their family members, family members became responsible for reminding them to take MMS. The delivery of MMS to the homes of participants was appreciated, as travel to DPHRU was viewed as cumbersome and costly, especially as many young women were unemployed.

#### Participant barriers to supplementation adherence

Four subthemes emerged when participants discussed barriers to MMS supplementation: knowledge of anaemia; lack of benefits of the MMS; side effects; and family support.

Some participants reported that they had not experienced any benefits from the MMS and hence did not feel motivated to continue with MMS and had since stopped supplementation. Some participants relayed that despite the information session at the onset of the intervention, they did not know why they were given MMS. Additionally, because of the unknown nature of the MMS, many participants concluded that any ailment they experienced during the intervention was caused by the MMS. For example, after experiencing an increase in acne during the intervention, one participant decided not to take the MMS, as she believed the MMS was the cause. Furthermore, the participants indicated that their community members did not take MMS, and the lack of understanding around supplements also caused some family members to be suspicious of the supplementation program. Those with family members who did not trust the MMS had stopped taking the MMS.

### Responses from CHWs

#### Barriers to supplementation adherence

Three subthemes were reported when CHWs discussed barriers to supplementation, which were: CHW knowledge of anaemia; community knowledge of supplements; and side effects. Quotes for these sub-themes are presented in Table 2.

**Table 2:** CHWs quotes regarding barriers and facilitators to supplementation adherence.

Barriers	Quote
**CHW knowledge of anaemia**	*‘I think there are so many notions about folic acid and iron in Soweto*, *the only time people hear about those two words is when they are pregnant in the clinic*. *So*, *the moment you mention those two words*, *people will assume that you’re trying to make them fertile and even the Health Helpers thought that*. *We needed to continue telling them and teaching them that it is not about pregnancy alone*, *it is about health issues in general–so nobody had an idea*, *you think it’s for pregnant people’*
	*‘I think it’s probably the container*, *it confused everyone*, *the fact that there was a pregnant woman on the container*, *it confused everyone like ‘oooo there’s a pregnant woman and what not*.*’*
	*I understood why we were giving supplements to anaemic*. *But I did not agree with giving supplements to the healthy’*
**Society knowledge of MMS**	*‘So then how do you say it’s a problem when I don’t even know about anaemia*. *Like high blood pressure and sugar diabetes*, *if you ask the young ladies we are dealing with*, *they know about it because someone in their house is dealing with that issue*. *They will tell you quickly*: *‘I know someone who has it and I know what it does to you*, *and they will explain but with anaemia it’s such a taboo term*, *nobody uses”*
**Side effects reported**	*‘Most of them have heavy (menstrual) flow*. *Most times its heavy flow and the okay*, *the other one is gaining weight*, *they eat a lot*.*’*
**Facilitators**	
**Access to MMS**	*‘It becomes more expensive; can you imagine if they travel to come and fetch their supplement*. *It’s going to become more difficult for them to get money to get there*.*’*
**Family support**	*‘Majority of families are supportive right*. *They just need you to explain what this is and why you are giving them this and what is happening and where you are from*. *If you explain yourself today*, *you’d meet the father*, *next time you meet the mother*, *you have to explain and next time you meet the grandmother*, *and the grandmother wants to know what is happening*.*’*

Some CHWs expressed that they understood why anaemic women received MMS but found it difficult to explain why non-anaemic women were receiving MMS. CHWs expressed concern about the MMS link to pregnancy, as well as how the lack of society knowledge around anaemia negatively impacted the intervention. CHWs recorded many reported side effects of the MMS, including an increase in menstrual blood flow and an increase in appetite leading to weight gain. They expressed experiencing difficulties dealing with the reported side effects. The increased appetite was of particular concern because the community was economically disadvantaged.

#### Facilitators to supplementation adherence

Two subthemes were reported when CHWs discussed facilitating factors of supplementation, which were: access, and family support.

Most of the CHWs expressed that the delivery system worked, as it ensured that the participants received the MMS. They felt that it would be costly for the participants to get the MMS from the unit. The CHWs relayed that it was crucial to obtain community and family trust when they worked with young participants. They reported that once a relationship of trust was developed, the family members became responsible for reminding participants to take MMS.

### Phase 2: Focus groups

#### Medication related beliefs

In Table 3, we show quotes on participant understanding and motivation for supplement use, participant concerns and emotions around medication, family and peer beliefs around medication, family and peer expectation and need for supplements, as well as family and peer beliefs around anaemia diagnosis. Most participants stated that they had little knowledge about anaemia before becoming a part of the trial, and participants reported that there was no vernacular term they knew that described anaemia. For some, hearing that they had anaemia, a ‘big word’, served as a motivator for their adherence. Some anaemic participants had heard nurses describe anaemia as ‘low blood’ as in reduction of blood in the body, which they associated with potential complications such as being pale, fatigue, tiredness, fainting, dizziness, and headache. However, the non-anaemic participants tended not to know any symptoms of having anaemia. Furthermore, the concept of taking supplements to prevent anaemia was not well understood by participants.

**Table 3:** Medication related beliefs.

Topic	Quotation
**Participant understanding and motivation for supplement use.**	*“Me I don’t think that for everyone it’s necessary to take iron supplements*, *because there are people like their immune system is strong like maybe they eat veggies*, *like people in the old days they didn’t get sick and they’ve never took supplements”*
**Participant concerns and emotions around the taking of medication**	*“Well*, *I don’t have much problem with medication*, *but I also think we need to go back in to being extra healthy cause if you are not extra healthy cause if you are not healthy you end up drinking a whole lot of medications or the western medication*. *So*, *I think we need to go back to our roots*. *I had to put it that way*.*”*
	*“Guys pills are not anyone’s favourite*, *if I were to choose*, *I wouldn’t take medicine*, *its tiring”*
**Family and peer beliefs around medication**	*‘I think there is a stigma around medicine*. *They fear being stigmatized*, *because growing up people with HIV were stigmatized a lot*, *so even sugar diabetes some people don’t even understand what sugar diabetes is*. *So*, *if you arrive carrying pills*, *what is it for they ask*? *…*.* *.* *. *People lack understanding of all the diseases out there*. *That’s why the youth is afraid to be stigmatized*.*’*
**Family and peer expectation and need for supplements**	“*OK*, *I have a cousin*, *right*? *chuckles* she doesn’t stay this side, *so she often comes this side to visit and every time I take my supplements she would say “you are sick*, *why are you always taking pills*, *no man; you are dying*!*”*
	“*OK the people that I’m close with believe in traditional medicines*, *so when you come with medicine from the doctor*, *they are not interested just like supplements; when I started taking them*, *they didn’t treat me well and they said I should stop taking them*…….”.”
**Family and peer beliefs around anaemia diagnosis and cures**	*“Previously I would even eat soil to cure my anaemia; and anything I come across hence I say that since I came here; I have seen that I have to eat like this and this but it’s a bit challenging*.*”*
	*“The only thing that… well*, *I think every black person says this you’re shocking*. *You don’t have iron*, *you’re shocking”*

Most of the participants reported that it was their first experience taking MMS, and that it was their first experience taking prescribed medication. Most anaemic participants cited that they understood why they had been given MMS. However, the participants who were non-anaemic at the baseline assessment did not think that the MMS were necessary. Additionally, most participants believed that MMS were medication, and medication in their opinion was taken when one had an ailment. However, though most participants trusted that medicine worked, some mentioned that using traditional methods–“going back to their roots” as they put it–could be more beneficial in curing disease. All participants said that they started taking the MMS. However, the majority stated that they did not like taking the supplements because they did not like medication. They equated supplements to medication.

Participants reported that because of the association between medication and the HIV pandemic, a stigma existed amongst community members with regards to young people taking medication. One participant reported that their family thought the MMS were “drugs,” while another discouraged MMS taking but instead encouraged her to use traditional medicines.

When asked about the knowledge that their families had on anaemia, most of the participants said their communities were not aware that anaemia was a health concern, neither did their communities know the symptoms or consequences of anaemia. Additionally, some participants reported that they had heard that being static when one touched steel objects caused by low iron levels in the blood. A few participants reported that community and family members had advised them to eat mud, or *Bar-one* chocolate and milk stout when they had presented with anaemia symptoms.

#### Medication related characteristics

In Table 4 we show quotes on perception of supplements taking routines, supplement characteristics, supplement adverse events and the healthcare system and supplements.

**Table 4:** Burden of medication related characteristics.

Topic	Quotation
**Burden of medication routine**	*“Have you noticed when you have stressed you think a lot of things at the same time there’s going to be that one that you forget and it’s going to be supplements and not that you did it on purpose your mind is all over the place then you think the time has just passed let me take the pills*
	*“Most of the time I don’t even know what day it is*, *so now I must remember to take supplements on a specific day*! *What day is it is difficult for me*, *so it’s easier from Monday to Sunday*.”
**Burden of medication characteristics**	*“I hate capsules*, *I just hate them for no reason*. *I just hate them*.*”*
	*‘” for me pills are not my favourite*, *to drink them I crush them with a plate and eat the powder*. *When I drink it alone it gets stuck”*
**Burden of adverse events**	*“Uhm*, *my side effects… okay*, *my side effects are when I started taking the pills it felt like I wanted to vomit so I would take water so can control the feeling and also after taking them I would start feeling dizzy and sleepy but the next day when I took them it felt better*, *I didn’t stop*, *now its treating me well Compared to the first time*, *I guess the body gets used to it*.*”*
**Burden of medication and the healthcare system- delivery system**	*‘mmhh*, *it’s nice*. *The fact that you don’t have to take a taxi and come collect*, *you just chill and they bring them*.*Ya uhm*, *my life is just too busy so I don’t*, *I wouldn’t have the time to be quite honest at all*.*’*
	*“I don’t think I was going to fetch them if I had to go to the clinic*. *Lines*! *Waking up in the morning*. *And going to the queues at 5; it’s too much; and since its COVID it’s even worse because you queue outside the gate till… and then they gradually let you in*.*”*
**Burden of medication and the healthcare system -relationship with trial**	*“Nurses these days don’t even examine a person like they used to*. *So here at least here*, *we get checked”*
	*“Uhm*, *I trust the supplements from here because here we have help officials*, *you see the ones from the clinic okay it fine*, *they usually give them to pregnant people and then the ones from SANBS they give to people after donating blood*. *They don’t even explain how they were made and all that*, *they just say we are giving out iron tablets after donating*, so the ones from here before they gave me ****** explained everything that’s why I say I trust the ones from here)”

Out of the twenty-six participants, only eight (anaemic participants) reported completing an uninterrupted course of MMS. The majority reported skipping doses or quitting. Participants on the bi-weekly regime cited difficulty in remembering days prescribed to take the MMS and this led to non-adherence. Others stated that the simple action of taking the MMS was tiring and adapting their lives to medication was an arduous task. Some participants reported that they were on other medication besides the MMS and having to take everything was burdensome.

Most participants complained about the characteristics of the MMS, such as the bad taste, the pills being too big, or having difficulty swallowing the MMS leading to non-compliance. One participant explained how her dislike of pills was linked to an unpleasant experience with medication. When asked about receiving the supplement in powder form instead of a capsule, some participants said it would be more appealing. The associated side effects were nausea, vomiting, dizziness, and headaches, which led some participants to stop taking them. However, a few participants developed coping skills and were able to continue supplementation despite side effects. Furthermore, many said that the challenges of life made it difficult to prioritise taking the supplement. The six months supplementation period was too long for some participants.

The participants mostly reported having a positive relationship with their CHWs. Information and support given by a CHW was viewed as motivational and helpful. However, some non-anaemic participants felt that information they were given by their CHW was insufficient. Additionally, the participants noted that the CHWs only spoke about anaemia at the first visit and when an assessment was done at six months. The sessions in between were not solely focused on anaemia, hence participants reported losing the impetus to continue with MMS and eating iron rich food. Participants mentioned that they preferred our CHWs to the government nurses, as CHWs took time to explain information to participants. Additionally, all the participants appreciated the delivery system, as it saved them time and was more convenient. Moreover, the participants appreciated that the MMS were free, and felt that this facilitated their adherence.

#### Suggested solutions from participants

Table 5 highlights participant quotes on possible solutions to improving the MMS intervention. Participants reported their need for family, CHW, and peer support to adhere to MMS. Their society members were seen as having limited knowledge on supplementation and community education was suggested. Women reported that having ambassadors in their communities for the MMS intervention would be helpful in explaining the benefits of MMS as well as promoting confidence in the supplements.

**Table 5:** Solutions from the participants.

Topic	Quote
**Family support**	*“Uhm*, *sometimes you might feel like not taking pills and you just feel like quitting but if you have support even at home if they remind you that don’t forget this and that*, *you get encouraged because you realize they care*, *and you don’t want to disappoint them so let me take them*. *It also shows that I can do it*. *Like I won’t fail taking pills*. *Put your differences aside*, *put your brave face on*, *life goes on*.*”*
**Peer group support**	*“If I had to be a Health Helper I would like*, *nothing beats competition*. *I would like to create a WhatsApp group with my participant’s ne*, *Okay*, *we all know our iron levels at first*, *then we set a common goal like whoever reaches here will win R5 airtime*. *People love something with an outcome*, *then you know if I continue drinking my supplement*, *I’ll get R5 airtime*. *So*, *I would encourage competition*, *nothing beats competition”*
**Support from Health Helpers**	*“Well*, *uhm okay*, *I think Health Helpers should follow up every day*, *like you don’t have to wait for the six months visit to communicate*, *call you participants there’s phones here you should call almost every day or send SMS’s or WhatsApp*.*”*
**Community engagement**	*“The same way they were advertising TB and HIV*. *Teach people*, *give people too much knowledge about anaemia and then teach them I think teaching someone who doesn’t know they will teach others and it goes on and on that how it will go that you know anaemia I heard about such person even that one will tell others”*
**The need to highlight benefits**	*“I think use people who have been already using them or people who use them*, *it treats them fine*, *you can use them to talk to people who you are giving supplements and they can say they gave me supplements; this is who I am*, *this supplement did this*, *this is how they treat me I think it would help*.*”*

## Discussion

This study has highlighted the complexity of administering MMS interventions in low-income settings. In phase one the identified facilitators to supplementation adherence were family support, CHW, access, experienced and perceived benefits. Reported barriers to supplementation adherence were family support, CHW knowledge on anaemia, lack of experienced benefits and side effects.

Family support was a key facilitator, in general, once family members understood the purpose of the MMS, they became supportive and played an integral role in reminding women to take the MMS. Several other studies have highlighted the importance of family support in aiding supplementation adherence [20–22]. Similarly, key family members, such as grandmothers, were reported to be significant barriers to supplementation adherence. The unknown nature of the consumption of supplements in the preconception phase, made some family suspicious of the MMS and hence they dissuaded the women from taking the MMS. These findings highlight the importance of engaging key family members in decisions surrounding anaemia prevention and treatment. It is not enough to engage only with women, outside of their family structures. Women’s decisions in our settings are greatly influenced by their families and cultures [23, 24].

In agreement with other studies, CHWs who were from the same community, of the same ethnic group and age played a major role in facilitating supplementation adherence [25–27]. The women viewed them as a helpful and reliable source of information on anaemia. However, since MMS are usually given to pregnant women, CHWs themselves were initially wary about giving the MMS to non-pregnant women in preparation for pregnancy. Further compounding the hesitancy of the CHWs, we found that they understood the concept of anaemia alleviation but did not fully understand the concept of anaemia prevention. Unfortunately, inadequate, conflicting, and sometimes contradicting information about iron supplements from health care providers is documented to negatively affect adherence [20, 28, 29].

Women in our study were largely unmarried, unemployed with some pursuing education; what is more, in their community, late marriage and economic independence of young women is generally viewed as highly desirable [30]. Sadly, the consequences of early pregnancy are numerous for these young women. Being a mother at this stage in their lives is related to an increased economic burden (raising money for the child), may lead to them dropping out of school, and is seen as a social burden, related to parental sanctions and time constraints for raising the child [30]. Therefore, providing them with the motivation that ‘MMS may help prepare women for pregnancy, which may lead to better birth outcomes’, was not viewed as a motivator; instead, it was a barrier to adherence. This although the pregnancy rate in South Africa is high. In 2012, a South African study showed that 62% of pregnancies amongst participants were unplanned, with about half ending in abortion [31]. Needless to say, that the packaging of the supplement with a picture of a pregnant woman, further hindered adherence. In addition to the unappealing link to pregnancy, the idea of supplementing for a probable future pregnancy seemed too ‘futuristic’ for the women. Hence, our participants like other women in other studies who experienced side effects, and did not see how supplements could improve their current lives, failed to implement coping mechanisms [32, 33].

Similar to a study by Galloway *et al* [20], women reported that because the MMS were administered in a capsule form, they viewed them as medication. Hence, in phase two we further unpacked how viewing supplements as medication affected adherence. We discussed the impact that individual, peer and family beliefs had on adherence, as well as the impact of medication characteristics, routine, adverse events and the health care system had on adherence.

It is documented that when women see iron containing supplements as medication, they in turn believe that supplements should only be consumed in response to specific symptoms and to ‘cure’ [34, 35]. This belief was detrimental to adherence, because those women who did not have any physical symptoms of anaemia as time progressed (or were taking the supplement for prevention) were not motivated to adhere to MMS, as they did not ‘need’ them. It is, however, possible to have anaemia with no apparent symptoms. Secondly, the dislike for ‘Western’ medication and the preference for ‘traditional’ methods to cure disease may have fuelled scepticism of the MMS [36–38]. Studies have shown that, one’s attitude towards medication has a significant effect on medication adherence [37–39]. Additionally, some women also mentioned that their dislike for MMS was linked to negative past experiences with medication. Mohammed *et al* [16] highlights in their review, that an individuals’ attitude towards medicines also plays a crucial role in persisting with the use of medicines. Further compounding the situation, the reported association of medication with the HIV stigma coupled with the community notion that young people do not need to take medication, could possibly have added a significant psychological burden to the women taking MMS [40, 41]. Additionally, women who were taking MMS in conjunction with other chronic medication, reported feeling overwhelmed. Likewise, in a study done by Ramalho-de *et al* [42] some patients related an increase in medicine number to a sense of loss of control and that negatively affected adherence. Moreover, the shape, taste and size of the MMS was unappealing to some participants and made them dread pill taking, and this as reported by other studies, may reinforce negative emotions towards medicine [36, 42, 43].

Like other studies [29, 32, 36, 44], some women cited that the routine of MMS taking interfered with their daily lives therefore, remembering to take it on the stated days was cumbersome and led to unintended non-adherence [33, 44]. Furthermore, other women reported forgetting to take MMS because they were consumed by their present challenges, and the immediate desire to improve themselves economically was their primary focus. Similarly, in the United Kingdom a study showed that threats related to youth’s economic and political environment had a negative impact on an individual’s health decision making capacity, resulting in young people deprioritising health related behaviour change [45].

As already reported in phase one, the CHW- participant relationship was a facilitator. Women reported that the CHWs were concerned about their welfare and took into consideration their personal lives when making decisions. The women stated that the MMS were free, and that our home delivery system made the women feel ‘special’ as well as was cost effective. It is well documented that the ease of a health care system promotes confidence and trust and may enhance adherence to medication [37, 39, 46].

**Fig 2:**
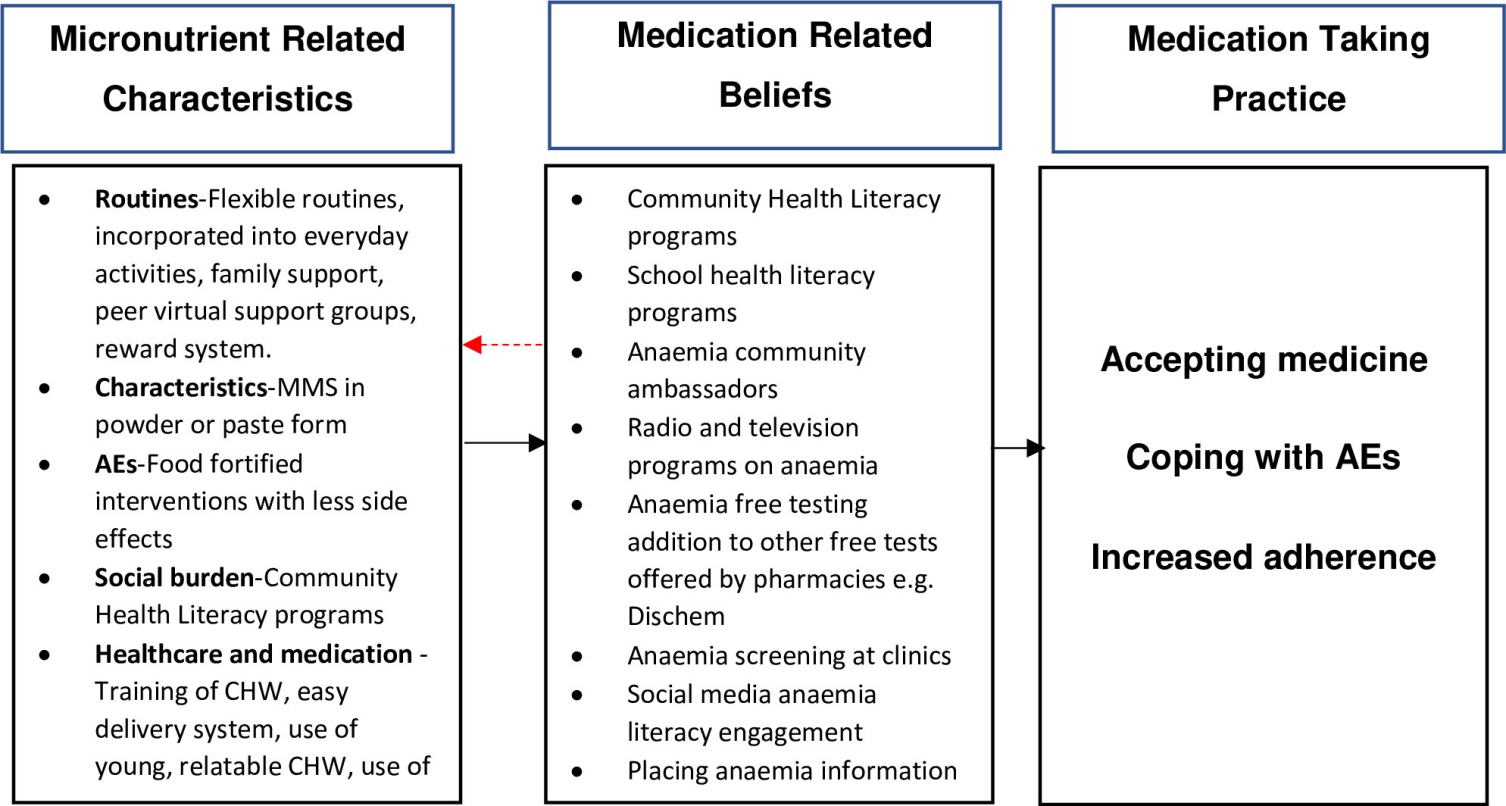
Conceptual framework on patients lived experience with MMS (adapted from Mohammed et al [16]) AEs adverse events.

Fig 2 below shows possible solutions to address these challenges. To be successful, preconception supplementation interventions need to adopt an approach that improves anaemia health literacy broadly in the community. Moreover, to enhance effectiveness of the intervention, the education must address intergenerational matters, such as complex mother-daughter relationships identified with young women in our income setting, which may negatively impact adherence [47].

To improve anaemia literacy we propose making use of the concept ‘treatment literacy’; which was coined in relation to the HIV pandemic in our health literacy programs [48]. This concept in our context, relates not only to building the capacity of women and their communities, to use MMS effectively but also to “interpret information about anaemia prevention, testing and care” and “prevent anaemia related stigma and discrimination” [49]. Examples of programs include television and radio information shows, adding anaemia literacy and tests to post-natal, contraception, and chronic medication visits at clinics, as well as free testing of anaemia at malls. To improve the anaemia literacy of young women we propose the addition of anaemia information on sanitary wear packaging, adding anaemia education to high school lessons, having anaemia education being done on social media websites such as Facebook and WhatsApp as well as using TikTok influencers to share information on anaemia.

To enhance effectiveness counselling of women should be based on Healthy Conversation Skills which includes women in decision making [50]. Counselling should highlight how the MMS may help them in coping with their present circumstance. For example, research has shown that reduction in anaemia prevalence among women not only improves maternal and child outcomes but also results in productivity gains because of improved physical capacity and increased cognitive ability [51]. The 2004 Copenhagen Consensus Panel estimated the benefit-to-cost ratio of iron interventions from resource savings; improvement in cognitive development and schooling, and physical productivity to be as high as 200:1 [52]. Furthermore, the MMS not only provides micronutrients of relevance in anaemia prevention/treatment, but they also have additional benefits, like strengthening the immune system, which could be an additional convincing current benefit [53]. We also suggest providing a supplement cover which highlights information on the benefits of supplementation (outside of pregnancy) [21, 22, 27].

To address the view of supplements as medicine, administering the supplements in powder or paste form may be beneficial [54]. To address routine challenges, as suggested by other studies, making use of a schedule that emphasises the option of taking the MMS when one remembers, rather than having a schedule that is viewed as rigid may improve adherence [55, 56]. Additionally, coupling supplement taking with everyday activities e.g., taking a supplement when you brush your teeth may help improve adherence.

CHW training, which is documented to improve supplementation [57], is needed to improve the CHW’s understanding about anaemia and the effects of supplementation in preconception phase. As the CHWs are concerned about the economic impact of MMS, their training should include information on the potential that preconception supplementation may have in improving the economic prospects of future generations [51]. When specifically asked what would help them adhere to MMS, the women mentioned that they would like more support. Surprisingly, the women expressed the desire to have peer groups, which were largely unsuccessful in our pilot study [58]. Nevertheless, as suggested by the participants, the use of virtual peer groups (Facebook and WhatsApp) could yield better results. Furthermore, the value of appointing peer MMS ambassadors (women who had used and experienced supplement benefits) to assist CHWs promote supplementation, was mentioned. In Ethiopia, the strategy of using young women who were successfully supplementing as motivators, supporters and educators of iron supplementation, improved behaviour towards iron supplementation among young women [59]. Moreover, the participants expressed a desire for frequent contact with the CHWs, where MMS issues could be reinforced. As proposed by Michie *et al*, continuously educating women may increase motivation to persist with interventions [60]. Finally, the women also drew attention to the power of a reward system, which is known to be beneficial in behaviour change [60]. Amongst our participants, financial rewards appeared to be a strong motivating force. Therefore, it may be worth considering the potential of a rewards programme in this setting.

The strengths of this study lie in the layered approach used to investigate the findings. The two phases allowed us to obtain a deeper understanding of the barriers and facilitators of preconception MMS supplementation. Additionally, the inclusion of the CHWs aided in further understanding the challenges of the intervention. The limitations of our study are that due to COVID-19 restrictions we were not able to do in-person interviews, and telephonic interviews were conducted instead. These telephonic interviews were short since women did not want to spend a long time on the phone.

## Conclusion

This study in non-pregnant women who are not consciously preparing for pregnancy but are likely to become pregnant, further builds on the existing conceptual framework for improving iron supplementation among women of reproductive age. Soweto is a setting, which like other in low- and middle-income countries, has numerous challenges for young women, which in turn influences their priorities and interaction with healthcare provision [58]. These competing interests highlight the need to develop supplementation programs that simultaneously address anaemia and their pressing current economic circumstances, e.g., embedding a job readiness program within the intervention, while highlighting the effects micronutrients may have on cognitive function. As the shift to preconception care (prevention) is novel, health care workers, women and their communities need to be thoroughly educated on its importance. Communities and women need to be educated on micronutrients and their role in disease prevention. Furthermore, instead of using the traditional antenatal care supplement packaging, rebranding maybe required to reflect the needs and benefits of MMS to non-pregnant women. Lastly the use of MMS ambassadors within communities could help promote confidence in MMS.

## Supporting information

S1 Text Focus group and interview guide questions. (DOCX)
