# Supplementary material for: Barriers and facilitators of micronutrient supplementation among non-pregnant women of reproductive age in Johannesburg, South Africa
Source: PLOS Glob Public Health. 2022 Nov 30;2(11):e0001310. doi: 10.1371/journal.pgph.0001310 (PMC10021408; doi:10.1371/journal.pgph.0001310)
Supplement: S1 Text — (DOCX) [file pgph.0001310.s001.docx]

**Focus Group Guide - Social Determinants of Iron Supplementation**

***Domain A-Structural Drivers***

1. Societal and cultural norms
   1. What does society/ your community consider to be medication?
   2. What is the purpose of medication in your community?
   3. If someone is known to be taking medication in your family, how are they viewed?
   4. What do you think your family and community know about anaemia? (is there a colloquial/traditional name given to anaemia)
   5. How is anaemia cured within your community?
   6. How are supplements in general viewed by your family?
   7. Are you aware of individuals who consume iron supplements and why they do so?
   8. What do you think your family and community understand about the use of iron supplements?
2. *Political circumstances-Question*
   1. How would you have responded if the supplements had been administered by the clinics or a government entity rather than us?

***Domain B – Nutrition issues***

1. What are nutrients?
2. In your opinion what is good nutrition?
3. What is the importance of nutrition in your everyday life? What is the importance of iron?
4. What are the sources of iron in your diet?
5. How can we obtain all the nutrients one needs? How can we obtain sufficient iron?
6. What challenges do you experience in attaining all nutrients?

***Domain C- Social position***

1. Education and communication
   1. How do you feel about taking medication?
   2. What previous experiences do you have taking medication?
   3. How do you feel about taking medication for an extended period? Can you tell us a time when you had to take medication for a month or more e.g., antibiotics……? (follow up question – did you take as prescribed…. elaborate)
   4. Can you please explain what supplements are?
   5. Can you tell me about a time when you took supplements before Bukhali trial?
   6. In your opinion what is the difference between supplements and pills(meds)?
   7. What is your understanding of anaemia?
   8. What are the symptoms of anaemia (how do you know you have it?).
   9. What do you think are the consequences/risks of having anaemia?
   10. What is the treatment for anaemia?
   11. Having had your iron levels assessed and explained to you. What do you think was the reason why you were given supplements?
   12. What do you think happens if you don’t take the supplements given to you as directed by the HH?
   13. When do you think it is appropriate for women to take iron supplements and why?
2. Socioeconomic circumstances
   1. How have your personal struggles (stress, anxiety) and challenges within your family/community affected your ability to take the supplements as prescribed by your health helper.

***Access***

What has your experience been regarding supplements being delivered to your home?

***Domain D- Circumstances of daily life***

1. Community and family social support
   1. How do your family members feel about you taking supplements from Bukhali?
   2. Do they understand why you are being given supplements?
   3. Explain what role your family plays in you adhering to the supplement regime?
   4. If you did not take it what role did your family play in, you not adhering to the supplement schedule?
2. Adherence (conceptual model of patients’ lived experience with PLEM)
   1. What motivated you to take the supplements for the Bukhali trial?
   2. What benefits have you experienced from taking the supplements for the Bukhali trial?
   3. How easy was it to take the supplement daily/weekly?
   4. How do you feel about taking supplements for an extended duration- every day for six months?
   5. Was there anything about the supplement itself that made it difficult for you to consume? How do you feel about the colour, taste, and size of the supplement?
   6. What side effects did you experience during the supplementation?
   7. How did you deal with the side effects?
   8. What other reasons do you have for taking supplements?
   9. What other reasons do you have for not taking the supplements?

Domain solutions

1. What information would be helpful for you and other young women to understand anaemia and the benefits of iron supplements?
2. How can we make iron supplements more acceptable in your community?
3. What can help young women to adhere to iron supplements?
4. If you were one of our HH, what would you do to encourage young women to take supplements?

**Interview Questions for Health Helpers**

1. What about being a health helper do you enjoy the most?
2. As a health helper you are responsible for facilitating the supplementation program. Can you tell me a positive experience that you have had with a participant in relation to the supplements?
3. What do you think makes it easier for a participant to take the supplements or buy into the micronutrient intervention?
4. In your opinion what have been the barriers that contribute to the participants not taking the supplements?
5. To what extent does the community or family of the participant contribute to the participant taking the supplements?
6. What are the common side effects that the participants have been bringing up?
7. How have they responded to the advice/ responses that you have given them?
8. What other challenges have you experienced in regard to the micronutrients?
9. What else do you think could make the micronutrient intervention more effective?
10. How else would you like to be supported in regard to the supplements?

Questions for Nicholas

1. What do you enjoy most about your job?
2. You have been distributing the supplements to our participants. Tell us when you had a good experience in delivering these supplements?
3. What has been your experience when trying to locate a participant e.g., when you call them and inform them that you would like to drop the supplements?
4. When you drop off the supplements and the participant is not available, how do the families/communities respond?
5. What makes it difficult for you to deliver supplements in some instances?

**Interview Questions for Participants**

Step 1 Perception Questions

1. How do you feel about your health?
2. What gets in the way of you prioritising your health?
3. What did your Health Helper tell you about why you were receiving supplements?
4. How did you feel about being given these supplements?
5. What do you think are the benefits of taking these supplements?
6. What benefits have you experienced from taking the supplements?
7. How did the supplements make you feel? (probe if they had any symptoms that they perceive to be side-effects of the supplements, and how bad these were)
8. What effect did the side effects have on you taking supplements further.

Step 2 Adherence Questions

1. What helped you remember to take the supplements?
2. How helpful was the calendar in reminding you to take the supplements?
3. What could be a more useful way to help you remember to take the supplements?
4. How do you feel about receiving a SMS to help you remember to take supplements?
5. How did your family /community feel about you taking the supplements?
